# Actinomycetes, an Inexhaustible Source of Naturally Occurring Antibiotics

**DOI:** 10.3390/antibiotics7020045

**Published:** 2018-05-24

**Authors:** Yōko Takahashi, Takuji Nakashima

**Affiliations:** Kitasato Institute for Life Sciences, Kitasato University, 5-9-1 Shirokane, Minato-ku, Tokyo 108-8641, Japan; takuji@lisci.kitasato-u.ac.jp

**Keywords:** actinomycetes, secondary metabolites, novel compounds, physicochemical screening, physical and chemical properties, structural diversity, biological activity

## Abstract

Global public health faces a desperate situation, due to the lack of effective antibiotics. Coordinated steps need to be taken, worldwide, to rectify this situation and protect the advances in modern medicine made over the last 100 years. Work at Japan’s Kitasato Institute has been in the vanguard of many such advances, and work is being proactively tailored to promote the discovery of urgently needed antimicrobials. Efforts are being concentrated on actinomycetes, the proven source of most modern antibiotics. We devised a novel physicochemical screening mechanism, whereby simple physico-chemical properties, in conjunction with related detection methods, such as LC/MS, LC/UV, and polarity, could be used to identify or predict new compounds in a culture broth, simply by comparing results with existing databases. New compounds are isolated, purified, and their structure determined before being tested for any bioactivity. We used lyophilized actinomycete strains from the Kitasato Microbial Library, most more than 35 years old, and found 330 strains were producers of useful bioactive substances. We also tested organisms found in fresh samples collected in the complex environments from around plant roots, as well as from sediments of mangrove forests and oceans, resulting in the discovery of 36 novel compounds from 11 actinomycete strains. A compound, designated iminimycin, containing an iminium ion in the structure was discovered from the culture broth of *Streptomyces griseus* OS-3601, which had been stored for a long time as a streptomycin-producing strain. This represented the first iminium ion discovery in actinomycetes. Compounds with a cyclopentadecane skeleton containing 5,6-dihydro-4-hydroxyl-2-pyrone ring and tetrahydrofuran ring, designated mangromicins, were isolated from the culture broth of *Lechevalieria aerocolonigenes* K10-0216 obtained from sediment in a mangrove forest. These structures are extremely unique among natural compounds. From the same culture broth, new steroid compounds, named K10-0216 KA and KB, and other new compounds having a thiazole and a pyridine ring, named pyrizomicin A and B, were discovered. New substances can be found from actinomycetes that have been exhaustively studied. Novel compounds with different skeletons can be found from a single broth of one strain. The sought after new antibiotics will arise from continued exploitation of the actinomycetes, especially rare actinomycetes. Work on new organisms and samples should be augmented by re-examination of known actinomycetes already in storage. New research should also be carried out on the manipulation of culture media, thereby stimulating actinomycete strains to produce novel chemicals. The establishment of wide-ranging international research collaborations will facilitate and expedite the efficient and timely discovery and provision of bioactive compounds to help maintain and promote advances in global public health.

## 1. Introduction

The use of chemicals to maintain or improve human health is as old as recorded history. In recent times, the discovery and development of chemicals to kill or overcome bacteria or pathogens is regarded as one of the most significant medical achievements of the 20th century, and countless millions of human lives have been saved as a result. Natural products have been and remain the mainstay of medical treatments. Chemicals produced in nature, or compounds based on them, accounted for 65% of the 1211 small molecule drugs approved by the United States Food and Drug Administration (FDA) in the 34 years from 1981 to 2014 [1]. The wide and diverse range of microbial primary or secondary metabolites that possess potent and sometimes unique bioactivity, coupled with the enormous and as yet relatively untapped potential and promise they offer, will heavily influence and drive forward future antibiotic research, while simultaneously emphasizing the importance of prioritizing natural products discovery over the manufacturing of synthetic compounds [2].

Penicillin was first used for human treatment in 1942, and it revolutionized the treatment of bacterial infections. It has since saved hundreds of millions of lives, as well as galvanizing the search for similar antibacterial or antimicrobial chemicals. As a result of the worldwide research effort, there was a flood of new antibiotics identified throughout the 1950s and 1960s, with the approval of several distinct novel classes of efficacious antibiotics for human use. However, since that “Golden Age”, the number of new antibiotics registered has steadily declined, and very few new classes of antibiotics have reached the marketplace and clinical use. In reality, scientific and economic factors will likely delay the appearance of any new antibiotics. From preclinical testing to approval for human use takes 10–15 years, and the costs involved are prohibitive [3,4]. A recent analysis suggests that, in 2014, the actual cost of driving a new compound from concept to the marketplace was in excess of $1.3 billion [5]. Furthermore, approximately 1 in 1000 potential drugs proceed to clinical trials, and then almost 90% fail in the human testing phase. For example, in the antibiotic field, the prevailing dangerous lack of new antibiotics, coupled with the loss of effectiveness of many already being widely used, threatens a return to the pre-antibiotic era, and the reversal of the gains made in global public health during the 20th century, accompanied by the potential loss of millions of lives.

The world is fast waking up to this dreadful scenario, and in 2015, the World Health Assembly endorsed the Global Action Plan on Antimicrobial Resistance [6]. This committed all member states to prepare national action plans and take proactive steps to promote the discovery, development, and sustainable exploitation of new antibiotics, especially those with novel modes of action. These commitments were reiterated by the United Nations General Assembly in 2016 [7].

So how can we find the urgently needed drugs? We firmly believe that actinomycetes will prove to be the primary source of the desperately needed biological substances over the next 2–3 decades, and this publication contains substantial evidence to support that point of view.

The actinomycetes are a heterogeneous group of Gram-positive bacteria with high guanine (G) and cytosine (C) content in their DNA. They are extremely diverse, with at least 350 genera known to date. They constitute one of the largest bacterial phyla, and are ubiquitous in aquatic and terrestrial ecosystems. Most (especially the streptomycetes are saprophytic, soil-dwelling organisms, but they are also found in fresh and salt water, and the air. They are typically present in soil at densities of 10^6^ to 10^9^ cells per gram of soil, with streptomycetes accounting for over 95% of all actinomycete strains isolated from soil [8]. Many species are harmless to animals and higher plants, while some are important pathogens.

The actinomycetes, particularly species from the genus *Streptomyces*, have proved to be a tremendous high-impact source of valuable chemicals. They have yielded many clinically essential antimicrobial compounds, including streptomycin, actinomycin, and streptothricin [8]. Besides streptomycin (discovered in 1944 from *Streptomyces griseus*), other examples of the success of this traditional discovery research approach are chloramphenicol (1947, *S. venezuelae*), tetracycline (1948, *S. rimosus*), erythromycin (1952, *Saccharopolyspora erythraea*), leucomycin (1952, *S. kitasatoensis*), and vancomycin (1956, *S. orientales)*. Additionally, in 1963, gentamicin was discovered, isolated from *M. purpurea*, a member of the *Micromonospora*. This triggered the search for new compounds from the so-called “rare actinomycetes”, which are lower frequency of isolation than members of the genus *Streptomyces* that are well isolated from soil. Compounds from the rare organisms include teicoplanin (1978, *Actinoplanes teichomyceticus*), fortimicin (1977, *M. olivoasterospora*), rosamicin (1972, *M. rosaria*), and nocardicin (1976, *Nocardia uniformis*). Incidentally, salinosporamide A, which holds promise for development of an anticancer drug, is produced by a strain of the genus *Salinispora,* a rare actinomycete isolated from a heat-treated marine sediment sample [9].

Approximately two-thirds of all known antibiotics are produced by actinomycetes, predominantly by *Streptomyces* [8]. It is believed that the actinomycetes are the source of some 61% of all microorganism-derived bioactive substances so far discovered [10], with 16% of the total originating from the “rare actinomycetes”, mostly from the *Micromonosporaceae*, with additional smaller contributions from the *Pseudonocardiaceae* and *Thermomonosporaceae*. This suggests that rare actinomycetes are a valuable source of novel compounds, and that improved isolation strategies are required to increase the frequency in which they are isolated [11].

## 2. Historical Discovery of Novel Compounds from Actinomycetes by the Kitasato Ōmura-Drug Discovery Group

The Kitasato Institute has, since its inception, concentrated its investigations on soil dwelling microbes, particularly the actinomycetes, as a potential source of bioactive compounds. Up until the mid-1970s, the singular universally employed discovery process involved identifying microorganisms in soil (or other) samples, culturing them and then testing any primary or secondary metabolites or other chemicals they produced to identify predetermined bioactivity that would meet a human need.

Decades of success in our exploration of the actinomycetes is exemplified by the discovery in the Kitasato Institute by Satoshi Ōmura in the early-1970s of *Streptomyces avermectinius* (synonym *S. avermitilis*) MA-4680^T^, the microbe which produces the avermectins [12,13,14]. The avermectin derivative, ivermectin, is perhaps the world’s greatest, most effective, and safest drug for the treatment and prevention of a diverse range of human diseases and conditions [15]. The importance and significance of the discovery and development of these compounds was recognized by the 2015 Nobel Prize in Physiology or Medicine being awarded to Prof. Ōmura and Prof. William C. Campbell of Merck & Company, Inc., Kenilworth, NJ, USA, representing the industrial partner which has become essential for the discovery, development, production, marketing, and distribution process of all modern-day antibiotics. The award citation stated “William C. Campbell and Satoshi Ōmura discovered a new drug, avermectin, the derivatives of which have radically lowered the incidence of River Blindness and Lymphatic Filariasis, as well as showing efficacy against an expanding number of other parasitic diseases” [16]. The 2015 award was the third Nobel Prize given for discovery of an antibiotic, following those for penicillin (for Fleming, Florey, and Chain in 1945) and for streptomycin (for Waksman in 1952), the man who first coined the term “antibiotic”.

The discovery of ivermectin arose because of Ōmura’s unwavering belief that microorganisms are a limitless source of useful chemical compounds—“Microbes do not produce useless metabolites: we just have little knowledge of their usefulness for mankind” [17,18], and because the partnership he set up between his group and Merck scientists were looking for specific anthelmintic compounds. Although ivermectin has proved to be a multifaceted, extremely effective chemical with a wide range of impacts, the original bioactivity screening focused almost predominantly on looking for an anthelmintic. Hence, that was what was found.

In the early-1970s, Ōmura decided to introduce an innovative new approach to drug discovery, namely to simply identify novel chemicals with no fixed goal in mind, carry out preliminary assays and evaluations, catalog and store both the chemicals and the producing microorganisms, and make the chemicals available for others to assay for all variety of bioactivity, or for use as biological or chemical reagents. This novel process was referred to as physicochemical (PC) screening.

As members of the Kitasato Institute for Life Sciences’ Drug Discovery Group, with Satoshi Ōmura as team leader, we have long and extensive experience in the search for novel compounds derived from microorganisms. Our cohesive integrated research program now encompasses the following three foci:

1. Isolation of microorganisms, identification, and microbial cultivation.
2. Discovery of substances from microbial metabolites.
3. Optimization of compounds by organic synthesis.

Latterly, our isolation work has been significantly refocused. We are now investigating existing but hitherto underutilized actinomycetes which exist in storage. We have also switched our attention from soil dwelling microbes to the exploration of microorganisms living in the complex environments found in the immediate vicinity of plant roots. We quickly discovered that whereas more than 90% of actinomycetes isolated from soil are *Streptomyces* strains, the rare actinomycetes dominate in strains isolated from plant roots. Currently, some 642 strains of actinomycetes have been isolated from 16 plant root locations, about 80% of which are rare. Two new genera (*Phytohabitans sufuscus* and *Rhizocola hellebori*) plus seven new species have, so far, been proposed through taxonomic study of these strains [19,20,21].

In our case, the discovery of useful microbial chemicals has been facilitated and accelerated by employing a two-pronged approach. Initially, using the traditional method attempting to acquire a new compound with a preconceived specific biological activity; more recently by identifying any and all novel substances by detecting and exploiting the basic physical and chemical properties and structural features of compounds. This bifurcated approach, both mechanisms of which are ongoing in Kitasato University, has led to the discovery of over 500 compounds, most of which were found using the original method [22].

In the mid-1970s, PC screening was introduced initially using Dragendorff’s reagent to identify nitrogen-containing compounds (alkaloids) which would cause a simple, visible color change. Staurosporine [23] was discovered as the first indolocarbazole compound from the culture broth of *Saccharothrix aerocolonigenes* subsp. *staurosporeus* AM 2282^T^ [24] (renamed *Lentzea albida* in 2002 [25]) in 1977, using this method. We initially determined that the compound possessed antifungal properties, and demonstrated a hypotensive effect. Nine years after discovery, in 1986, another research group discovered that staurosporine was a nanomolar inhibitor of protein kinases, as assessed by the prevention of ATP binding to the kinase [26,27]. This interesting biological activity stimulated an explosion in exploratory research for selective protein kinase inhibitors by numerous laboratories and pharmaceutical companies worldwide, staurosporine becoming the parent compound for many of today’s highly-successful anticancer agents. This example helps to illustrate that all substances produced by microbes may be of great benefit, and that they should be examined for potential use in all forms of human endeavor, especially for use in modern medicine, and that they should be made available for exhaustive testing and use by all, wherever practical and possible.

We now routinely search for novel chemicals from actinomycetes by analyzing a range of physico-chemical properties, such as LC/MS, LC/UV and polarity. It is now possible to predict whether a new substance is present by analyzing results and comparing with existing databases. This approach has so far identified some 36 novel compounds (including analogs) [28]. In this report, we describe these results, and discuss the ability of actinomycetes to produce a wide spectrum of novel chemicals, as well as draw attention to the diversity of metabolites that a single microbial strain can provide for us.

## 3. Novel Compounds Discovered by Physicochemical (PC) Screening of Cultured Broths of Actinomycetes

The novel compounds derived from actinomycetes discovered through our PC screening during the past eight years are displayed in Table 1. The compounds are accompanied by the name of the producing microorganism, their original source, the primary biological activity of the compound and relevant publications. The PC screening procedure was carried out as follows.

After cultivation in 10 mL of several kinds of preset media, an equivalent amount of ethanol was added, the ingredients were thoroughly mixed, the cells were then disrupted, and the ethanol extract was subjected to PC screening.

After LC/MS and LC/UV analysis, each peak recorded was compared with known data from the Dictionary of Natural Products, and our own database. A peak was predicted to be a novel substance. When this was the case, we scaled up the culture and isolated and purified the target compound using column chromatography and preparative HPLC. After obtaining the unique compound, its structure was determined by high-resolution mass spectrometry, NMR, etc. The new compounds underwent preliminary bioassays, either in-house or in established collaborative research projects with other groups.

Identification of the strains being cultured was carried out using morphological characteristics, chemical composition in cells, and phylogenetic analysis based on 16S rRNA gene sequences.

The Kitasato Ōmura-Drug Discovery Group has already discovered avermectin [14], staurosporine [23], herbimycin [29], setamycin [30], and lactacystin [31] from secondary metabolites of actinomycetes [22]. The actinomycete strains producing these compounds (as well as strains producing a variety of other chemicals) have all been catalogued, freeze-dried, and stored in the Kitasato Microbial Library (KML). In an effort to respond to the urgent global demand for new antibiotics, we have recently revived the KML strains to confirm their viability, the continuance of compound production, and the reliability of the preservation process. Survival rates and compound productivity maintenance rates have been good, but specific data in this respect will be reported elsewhere. During this work, PC screening was carried out on culture broths of 330 strains, resulting in the discovery of several new compounds (No. 1 to No. 3 in Table 1).

With respect to the three entries in question, the name of the original compound and retention period by lyophilization are stated, all three having been stored for 35 years or more. The compounds recently discovered, namely bisoxazolomycin [32], the iminimycins [33,34], and the nanaomycins [35,36], would probably not have been detected by an assay system seeking a specific bioactive property, the compounds being found as a direct result of PC screening. Discovery of the iminimycins and nanaomycins are described in detail below in Section 4.2.

With respect to new isolates (No. 4 to No. 13 in Table 1), actinomycete strains isolated from around the roots of plants (No. 4 to No. 7), sediment from mangrove forests (No. 8 to No. 10), sea sediment (No. 11), and soil samples (No. 12 & No. 13) are listed. Actinoallolides [37], hamuramicins [38], spoxazomicins [39,40], and trehangelins [41,42] were discovered from endophytic actinomycete strains, and these are classified as rare actinomycetes. The mangromicins [43,44,45], K10-0216 KA and KB [46], and pyrizomicins [47], which have differing core structures, were found in a culture broth of *Lechevalieria aerocolonigenes* K10-0216 isolated from sediment from mangroves. Mumiamicin [48] was found in an actinomycete strain isolated from sea sediment, while sagamilactam [49] and the dipyrimicins [50] originated in actinomycete strains from soil. In Section 5.1, we describe, in detail, the discovery of other compounds, notably the trehangelins from *Polymorphospora rubra* K07-0510 and compounds from *Lechevalieria aerocolonigenes* K10-0216.

Assays of the 36 compounds, involving collaboration with other research groups, led to the discovery of varying bioactivity, as shown in Table 1. These results help demonstrate the usefulness and cost/time effectiveness of PC screening, as well as the potential diversity of metabolites produced by a single microorganism. The outcome clearly demonstrates that, as Prof Ōmura rightly opines, “microorganisms are a treasure trove of new natural products”.

## 4. Novel Compounds Discovered from the Kitasato Microbial Library (KML)

### 4.1. Iminimycin A by Streptomyces griseus OS-3601, a Streptomycin Producing Strain

**Figure 1:**
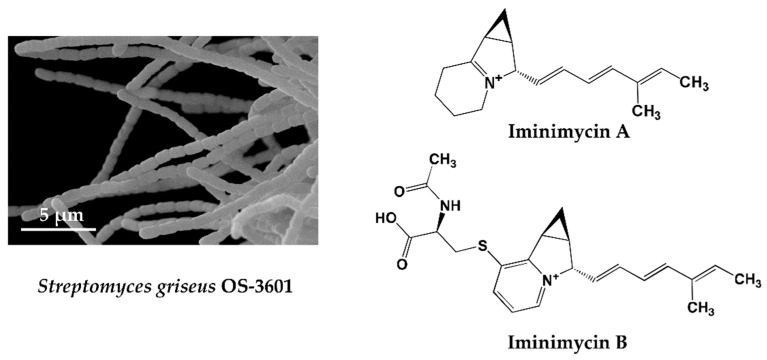
Scanning electron micrograph of the aerial spore chain of the streptomycin-producing strain *Streptomyces griseus* OS-3601 (**Left**) and structures of iminimycin A and B discovered from the culture broth (**Right**).

KML strain OS-3601 (Figure 1) was isolated from a soil sample collected at Aso, Kumamoto prefecture, Japan, in 1981, and identified as *Streptomyces griseus*, which produces streptomycin. As part of our screening of 330 preserved actinomycete strains, using four different liquid production media, a unique metabolite, predicted to be a new compound, was observed in an extract of a culture of this strain grown on defatted wheat germ medium. The metabolite was not observed following growth in the other three production media. The compound produced by strain OS-3601 showed an 242.1910 *m/z* [M + H]^+^ and maximal absorption at 269 and 282 nm. Purification from the culture broth of strain OS-3601 yielded a new iminium compound, designated iminimycin A (Figure 1) [33], which was strongly supported by an IR absorption spectrum, indicative of the presence of an iminium ion. Several plant-derived compounds containing an iminium ion are known [51] but, to our knowledge, this is the first compound arising from an actinomycete source.

An unidentified compound with physical and chemical properties similar to iminimycin A was observed in the octadecyl silyl fraction lacking iminimycin A; the compound having an 399.1736 *m/z* [M + H]^+^ and maximal absorption at 269 and 282 nm. Purification of this compound revealed a new indolizine alkaloid, designated iminimycin B (Figure 1) [34], that possessed *N*-acetylcysteine and pyridinium moieties, instead of the iminium moiety of iminimycin A. Iminimycin A and B both show antimicrobial activity against Gram-positive and Gram-negative bacteria. Since 1943, when Selman A. Waksman discovered streptomycin from the secondary metabolite of *Streptomyces griseus*, about 200 compounds have been reported from strains identified as *Streptomyces griseus* [9].

Among the actinomycetes, *Streptomyces griseus* is one of the organisms most frequently isolated from soil samples, and has been studied extensively. It was therefore surprising that our PC screening allowed us to find new substances from this strain, demonstrating, yet again, the unmatched ability of microorganisms, especially actinomycetes, to produce a plethora of chemicals.

One chemical component of the culture broth of strain OS-3601 was predicted to be novel from analysis of HPLC and LC/MS data gathered through PC screening. Our prediction proved true following isolation, purification, and structure determination.

Mapping of the genome of streptomycin-producing *Streptomyces griseus* has already been completed [52], and this information also indicates that production of the novel substance is predictable. So why has it not been discovered before? It is conceivable that productivity is low, or that it has been overlooked because it has been masked by the cycloheximide produced in large quantities at the same time in a lipid soluble fraction, or because the compound is inherently unstable and short-lived. We believe that prediction of the existence of new compounds can be envisaged through a combination of an enquiring mind, technological progress, and the creation and use of more sophisticated equipment. Indeed, it can be said that Prof. Ōmura’s belief that “microorganisms are infinite resources” and that “microbial research has really just begun” are an accurate depiction of the current situation.

### 4.2. Nanaomycins F, G and H Derived from Nanaomycins A–E Produced by “Streptomyces rosa subsp. notoensis” OS-3966

**Figure 2:**
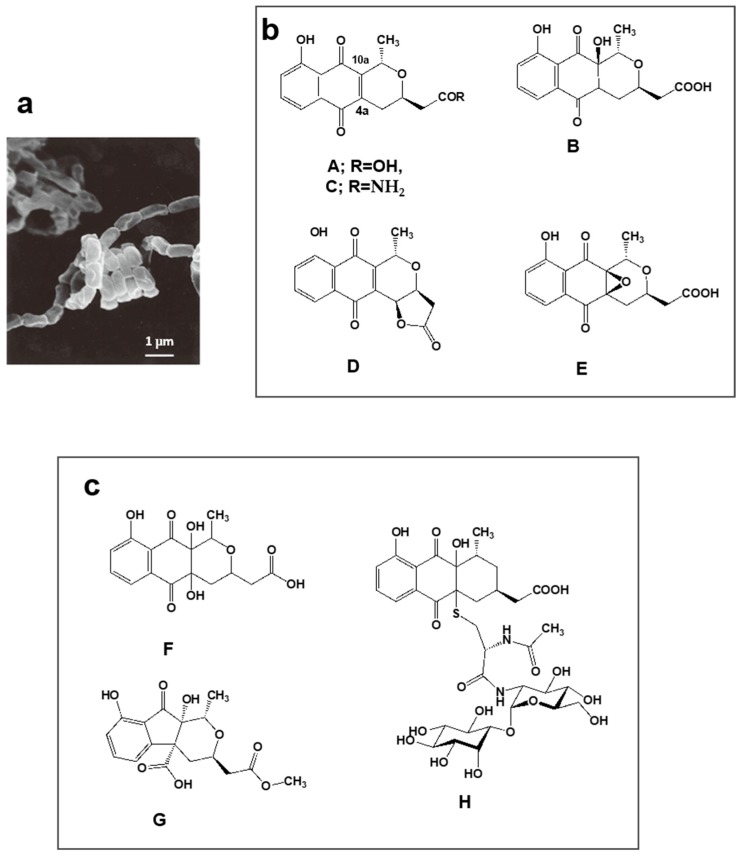
Nanaomycin-producing strain “*Streptomyces rosa* subsp. *notoensis*” OS-3966 and new analogs discovered by PC screening from a culture broth. (**a**) Scanning electron micrograph of aerial spore chain of “*S. rosa* subsp. *notoensis*” OS-3966 grown on agar medium; (**b**) Nanaomycins A–E discovered as antibacterial and antifungal substances during 1974–1979; (**c**) New analogs (F–H) discovered from the culture broth via PC screening.

Nanaomycins A, B, C, D, and E produced by “*Streptomyces rosa* subsp. *notoensis*” OS-3966 (Figure 2a,b) [53,54,55] were isolated from soil sampled in Nanao City, Japan. The nanaomycins contain a naphthoquinone skeleton and were found to have antimycoplasma properties. Nanaomycin A (Figure 2b) was developed as a therapeutic agent for cattle dermatophytosis in 1980 [56]. It generates semiquinone radicals by forming double bonds at positions 4a and 10a, damaging DNA in the process, consequently displaying antibacterial and antifungal activity.

We undertook PC screening using the culture broths of stored freeze-dried nanaomycin-producing strain OS-3966. Two new compounds appeared in the EtOH extract obtained from culturing in defatted wheat germ production medium. The first compound had 337.0917 *m/z* [M + H]^+^ and maximal absorption at 231, 248 (sh), 267 (sh), and 347 nm; the second compound had 351.1075 *m/z* [M + H]^+^ and maximal absorption at 225, 256 (sh), and 308 nm. Purification of these compounds revealed they were two new nanaomycin analogs. The first, which we named nanaomycin F (Figure 2c) [35], is a 4a-hydroxyl analog of nanaomycin B (Figure 2b). The second, which we named nanaomycin G (Figure 2c) [35], has a unique 1-indanone skeleton fused with a tetrahydropyran ring. During chromatographic purification of these compounds, another new compound was obtained, nanaomycin H (Figure 2c) [36]. Structure elucidation of nanaomycin H showed it to be a pyranonaphthoquinone with a mycothiol moiety. Our assays detected no antibacterial or antifungal activity in these three compounds. The reason for this seems to be that the radical-generating ability disappeared due to the reduction of the double bond of the quinone skeleton. It was found that the compounds inhibited epithelial-mesenchymal transition, inducing proliferation of mammalian cells [57]. These compounds would not have been found using an assay simply targeting bioactivity. Incidentally, the production medium of nanaomycin A to E is mainly composed of glycerol and soybean meal [53], whereas nanaomycins F, G, and H were produced on a medium containing mainly soluble starch and defatted wheat germ [35,36]. This raises the interesting possibility that simply changing the composition of culture media may allow the discovery of new compounds. These results showed to support OSMAC (one strain, many compounds) approach [58].

## 5. Novel Compounds Found from Fresh Isolates

### 5.1. Novel Substances, Trehangelins Found from Metabolites of the Plant-Derived Rare Actinomycete Polymorphospora Rubra K07-0510

Microbes isolated from plant root environments were cultured in each of four production media, with the resulting broths being subjected to PC screening. Strain K07-0510, when grown in one of the production media, yielded a peak (indicating a new compound) that was predicted based on spectrometric data.

**Figure 3:**
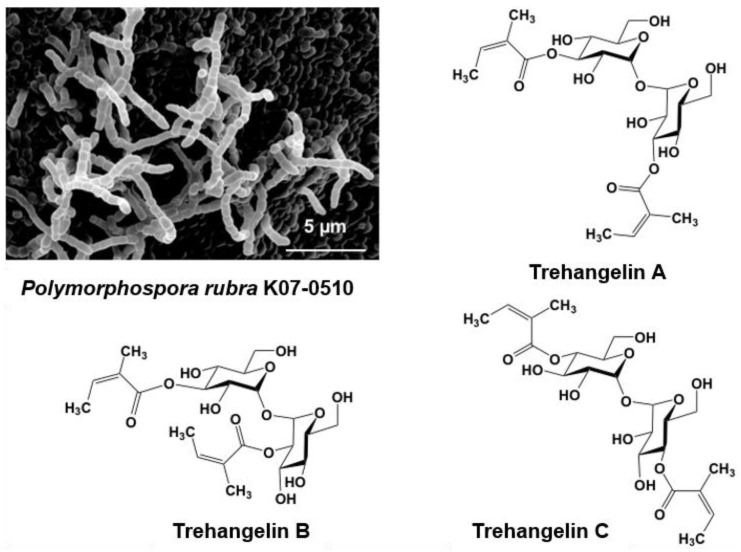
Scanning electron micrograph of the aerial spore chain of the trehangelin-producing strain *Polymorphospora rubra* K07-0510 and structures of trehangelin A, B, and C.

Strain K07-0510 was isolated from the roots of an orchid collected on Iriomote Island, Okinawa, Japan. Short spore chains were formed, and spores with a smooth surface were cylindrical in shape (Figure 3). Whole-cell hydrolysates contained *meso*-DAP (diaminopimelic acid). The 16S rRNA gene sequence was determined and analyzed using the EzTaxon-e database (present name EzBioCloud) [59] to reveal a 99.9% similarity with *Polymorphospora rubra* TT97-42^T^. On the basis of the morphological and cultural properties and 16S rRNA gene sequence analyses, strain K07-0510 was identified as *Polymorphospora. rubra*.

A new predicted peak showing 507.2087 *m/z* and maximal absorption at 216 nm was also found in the culture broth of strain K07-0510. Purification of this compound eventually identified three new compounds, which were named trehangelin A, B, C (Figure 3) [41,42]. These compounds were separated from the culture broth of strain K07-0510 by ethyl acetate extraction, followed by silica gel and ODS column chromatography, with final purification by HPLC. Eighteen liters of culture broth yielded 59 mg of trehangelin A as the major component, along with 4.4 mg and 1.3 mg of the minor components trehangelin B and C, respectively.

Structural analysis revealed that two molecules of angelic acid were bound to one molecule of trehalose. Trehangelin A, the main compound, binds angelic acid to the 3,3′ positions of trehalose, while B and C does so to the 3,2′ and 4,4′ positions, respectively. After substance isolation, preliminary bioassays were carried out, and it was found that trehangelin A and C inhibited erythrocyte hemolysis by photooxidation. Trehangelin A and C, which have symmetric structures, showed more potent inhibition than ascorbic acid. In addition, it has been found that the compounds facilitate cytoprotective action and accumulation of procollagen type I C-peptide in a cell culture assay [60], and further research is currently in progress. Our group is now close to elucidating the genetic basis of the biosynthesis of the trehangelins [42].

Angelic acid is known to occur in many plants, particularly chamomile, and has been used as a tonic and sedative to treat a variety of minor complaints, but reports of it being produced by microorganisms are extremely rare. It is, thus, interesting to note that a microbe in a plant root environment also produces the compound.

### 5.2. New Compounds Produced by Lechevalieria Aerocolonigenes K10-0216, Mangromycin A-I, K10-0216 KA & KB, and Pyrizomicin A & B

**Figure 4:**
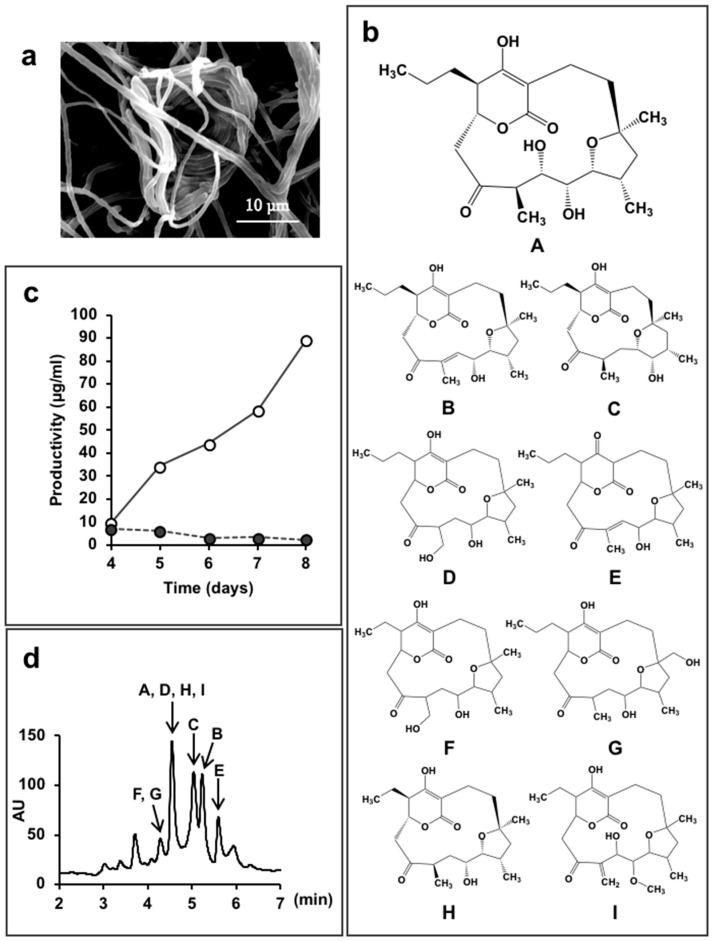
*Lechevalieria aerocolonigenes* K10-0216 and the mangromicins discovered from the culture broth. (**a**) Scanning electron micrograph of a clump of interwoven hyphae of *L. aerocolonigenes* K10-0216 grown on inorganic salt–starch agar at 27 °C for four weeks; (**b**) Structure of mangromicin A–I; (**c**) Productivity of mangromicin A. Black circle: Basic medium; soluble starch 2.0(%), defatted wheat germ 1.0, glycerol 0.5, dry yeast 0.3, CaCO_3_ 0.5, meat extract 0.5. Open circle: Improved medium; soluble starch 5.0(%), defatted wheat germ 1.0, glycerol 0.5, dry yeast 1.0, CaCO_3_ 0.5, meat extract 0.0. (**d**) HPLC analysis of the mangromicins. Chromatographic separation was undertaken using a MonoBis (3.2 × 150 mm, Kyoto Monotech Co., Ltd., Kyoto, Japan) at 40 °C. With regard to gradient elution, solvent A was water with 0.1% formic acid, and solvent B was methanol with 0.1% formic acid. The gradient elution was 0–10 min and 5–100% B. The flow rate was 0.5 mL/min, the injection volume was 5 μL, and detection occurred at 254 nm using a photodiode array detector.

*Lechevalieria aerocolonigenes* K10-0216 (Figure 4a) is a rare actinomycete isolated from sediment collected in a mangrove forest in Iriomote Island, Okinawa Prefecture, Japan in 2011. In the culture broth of this strain, we found 13 new compounds.

Mangrove trees growing in brackish water are known to be a rich source of microorganisms, and many rare actinomycetes have been discovered in such environments [61]. We isolated 65 actinomycetes strains from five samples collected from a mangrove forest. Simple use of 16S rRNA gene sequences resulted in identification of 44 strains of the genus *Micromonospora*, as well as a few from the *Actinomadura* and *Verrucosispora*. The so-called rare actinomycetes accounted for 83% of organisms isolated.

Strain K10-0216 grown on an inorganic salt–starch agar medium produced sparse white aerial mycelia that formed characteristic clumps of interwoven hyphae, as shown in Figure 4a. The 16S rRNA gene sequence, which was determined and analyzed using the EzTaxon-e database [59], demonstrated a 99.8% similarity to *Lechevalieria aerocolonigenes* ISP 5034^T^. On the basis of the morphological and cultural properties and 16S rRNA gene sequence analyses, strain K10-0216 was identified as *Lechevalieria aerocolonigenes*.

We found that the culture broth contained predicted novel substances with molecular weights of 410 or 392, and maximal absorption at 251 nm or at 236 nm, respectively. We named these compounds mangromicin A and B [43]. However, these peaks were not obtained in a jar fermenter culture. A total of 500 mL Erlenmeyer flasks containing 100 mL culture medium were used to obtain the target substances that were isolated and purified. Structural analysis revealed a unique structure of a cyclopentadecane skeleton containing a 5,6-dihydro-4-hydroxyl-2-pyrone ring and a tetrahydrofuran ring (Figure 4b). Both substances displayed antitrypanosomal activity, and a patent application was filed. Subsequently, we experimented with different production media in order to obtain a large amount of the target compounds. As a result, by changing the concentration of soluble starch in the medium ingredients from 2.0% to 5.0%, and dry yeast 0.3% to 1.0%, the amount of mangromicin A increased dramatically, from 0.24 μg/mL to 88.6 μg/mL (Figure 4c). Eventually, nine analogs were obtained from 15 L of culture solution using this procedure (Figure 4b) [44,45]. The HPLC profile is shown in Figure 4d. After isolation and purification, preliminary bioassays identified DPPH radical scavenging activity and, in particular, NO scavenging was potent [44,45].

**Figure 5:**
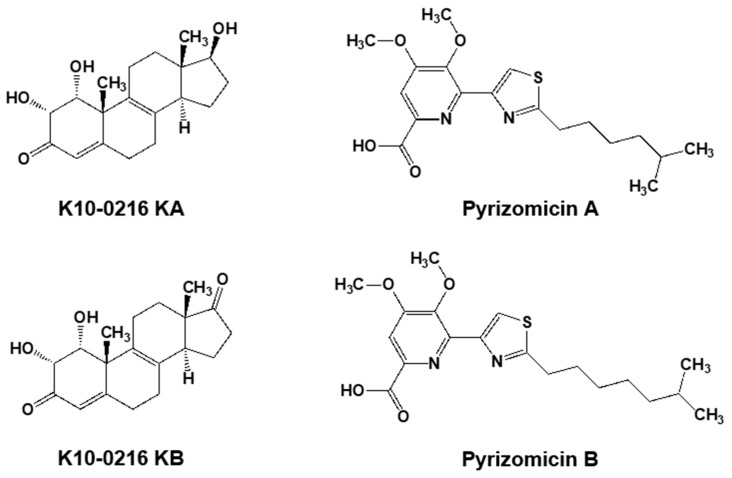
Two steroid compounds (K10-0216 KA and KB) and two compounds containing thiazole and pyridine (pyrizomicin A and B) from *L. aerocolonigenes* K10-0216 (Figure 4a).

Furthermore, from the same culture broth, we also discovered four novel compounds (Figure 5). Two contained a steroid skeleton (named K10-0216 KA and KB) that inhibited lipid accumulation in 3T3-L1 adipocytes [46], while the others displayed a thiazole and a pyridine ring (named pyrizomicin A and B) that showed antibacterial activity [47].

As described above, 13 compounds, including three with different skeletons, were found from a single culture broth of one microorganism. This reinforces our belief that it is highly likely to be able to identify and acquire new compounds simply by changing the culture method and production medium.

## 6. Conclusions

This report describes compounds derived from actinomycete strains which were found via PC screening between 2011–2017 by the Kitasato Ōmura-Drug Discovery Group. Iminimycins and nanaomycins were found from culture broths of conserved KML strains, while the trehangelins from *P. rubra* K07-0510 and new compounds, mangromicins, K10-0216 KA and KB, pyrizomicin A and B, from *L. aelocolonigenes* K10-0216, originated in fresh isolates. Our results illustrate that PC screening can unearth novel compounds that are not likely to be discovered through traditional targeted bioassay systems. Genomic analysis of actinomycetes has found that more than 30 secondary metabolite biosynthetic genes may be involved in chemical production, depending on the strain. However, knowing the genetic production mechanism may not necessarily make obtaining the compound easier.

This report also shows that it is possible to obtain a novel compound from actinomycete species, such as *Streptomyces griseus*, that has already undergone long and intensive study. In addition, as in the case of *L. aerocolonigenes* K10-0216, a variety of compounds with significantly different skeletons can be obtained from a single culture broth using only one microbe strain.

Our work also demonstrates that the aptly classified “rare actinomycetes” remain a unique and, so far, relatively unexploited source of potentially useful chemicals. This is supported by work that found that strains of the genus *Actinoallomurus* (a rare actinomycete) have high secondary metabolite production capacity [62]. We also found five actinoallolide analogs [37] (shown in Table 1) produced by *A. fulvus* MK 10-036 and *A. fulvus* K 09-0307, later discovering two more new compounds together with seven known compounds from an *A. fulvus* K09-0307 broth.

Microorganisms can present us with interesting compounds with unique structures which our current scientific knowledge and expertise cannot easily predict nor easily replicate. For example, the mangromicins have unique skeletons which subsequently attracted attention in the field of organic synthesis. Organic chemists tried to devise a total synthesis of mangromicin A, and finally managed to achieve it via a complicated and lengthy 30 step process [63], whereas *L. aerocolonigenes* K10-0216 produces the compound naturally during culturing.

To illustrate the immeasurable scope for success in this respect, it has been reported that 99% of microorganisms in nature have not yet been isolated [64]. Furthermore, our results suggest that interesting compounds can be found even from *Streptomyces* strains that are thought to have been exhausted, simply by devising new identification methods or culture conditions. It is therefore essential to revisit existing actinomycetes, common and rare, and comprehensively examine them for new compounds.

Traditionally, the search for useful natural products has been advanced using approaches that focus on specific biological activity and target molecules. In this approach, there is a clearly defined goal. However, with this method alone, it is likely that many of the microorganisms isolated have been exploited and then discarded without fully utilizing their abilities. It is clearly difficult to devise a wide variety of screening systems and, certainly, almost impossible for all these systems to be operational in a single institution. Consequently, extensive, multifaceted research collaborations will need to be established to work towards full and comprehensive testing of all chemicals, be they newly isolated from existing compound libraries or from new sources. Naturally, many obstacles will need to be overcome, including protection of Intellectual Property Rights, transfer of technology, and capacity building aspects. None of these should be insurmountable, especially so if the goal of getting as many new bioactive substances in the shortest time possible is to be achieved.

We remain firm in our commitment to discover as many new compounds as possible by exploiting the ability of microorganisms, especially the actinomycetes, to produce such attractive substances. Moreover, we will continue to follow a twin-pronged approach to this task, while striving to devise other alternative screening methods, and adopt any other measures that could help expedite the research and development process.

## Figures and Tables

**Table 1:** Compounds discovered by physicochemical screening of actinomycete strains (March 2018).

No.	Compound	Producing Microorganism	Source	Biological Activity	References
1	Bisoxazolomycin	*Streptomyces subflavus* subsp. *irumaensis* AM-3603	* KML Irumamycin producing strain, ** 36 years	Antibacterial	[32]
2	Iminimycin A & B	*Streptomyces griseus* OS-3601	* KML, Streptomycin producing strain, ** 43 years	Antibacterial	[33,34]
3	Nanaomycin F–H	“*Streptomyces rosa* subsp. *Notoensis*” OS-3966	* KML Nanaomycin producing strain, ** 36 years	Inhibitor of Epithelial-Mesenchymal Transition induced cells	[35,36]
4	Actinoallolide A–E	*Actinoallomurus fulvus* MK10-036	Roots of *Capsicum frutescents* in Thailand	Antitrypanosomal	[37]
		*A. fulvus* K09-0307	Roots of mondo grass in Saitama Pref., Japan		
5	Hamuramicin A & B	*Allostreptomyces* sp. K12-0794	Roots of fern in Hamura city, Tokyo, Japan	Antibacterial	[38]
6	Spoxazomicin A–C	*Streptosporangium oxazolinicum* K07-0460^T^	Roots of orchid in Iriomote Island, Japan	Antitrypanosomal	[39,40]
7	Trehangelin A–C	*Polymorphospora rubra* K07-0510	Roots of orchid in Iriomote Island, Japan	Anti-Lipid peroxidation	[41,42]
				Enhanced production of collagen	
8	Mangromicin A–I	*Lechevalieria aerocolonigenes* K10-0216	Sediment from mangrove forest in Iriomote Island, Japan	Antitrypanosomal	[43,44,45]
				Antioxidative	
9	K10-0216 KA & KB	*Lechevalieria aerocolonigenes* K10-0216	Sediment from mangrove forest in Iriomote Island, Japan	Inhibitory effect on the lipid accumulation	[46]
10	Pyrizomicin A & B	*Lechevalieria aerocolonigenes* K10-0216	Sediment from mangrove forest in Iriomote Island, Japan	Antimicrobial	[47]
11	Mumiamicin	*Mumia* sp. YSP-2-79	Sea sediment, Namako Pond in Kagoshima Pref., Japan	Antibacterial	[48]
				Antioxidative	
12	Sagamilactam	*Actinomadura* sp. K13-0306	Soil, Kanagawa Pref., Japan	Cytotoxicity	[49]
				Antitrypanosomal	
13	Dipyrimicin A & B	*Amycolatopsis* sp. K16-0194	Soil, Okinawa Pref., Japan	Antibacterial	[50]

* KML: Kitasato Microbial Library, ** length of preservation by lyophilization; No. 1–3: Compounds from the KML; No. 4–13: Compounds from fresh isolates (No. 4–7; Roots of plants, No. 8–10; Sediment of mangrove forest, No. 11; Marine sediment, Nos. 12 & 13; Soil).
