# Correction: Yōko, T., et al. Actinomycetes, an Inexhaustible Source of Naturally Occurring Antibiotics. *Antibiotics* 2018, *7*, 45

**DOI:** 10.3390/antibiotics7030074

**Published:** 2018-08-14

**Authors:** Yōko Takahashi, Takuji Nakashima

**Affiliations:** Kitasato Institute for Life Sciences, Kitasato University, 5-9-1 Shirokane, Minato-ku, Tokyo 108-8641, Japan; takuji@lisci.kitasato-u.ac.jp

The authors wish to make the following corrections to this paper [1]:

## 1. Changes in Main Body Paragraphs

There are some mistakes in this article [1]. Ref. 8 in line 47 of page 2 should be ref. 9; ref. 9 in line 6 of page 3 should be ref. 10; ref. 10 in line 9 of page 3 should be ref.11; ref. 11 in line 13 of page 3 should be deleted; ref. 12–14 in line 24 of page 3 should be ref. 12,13; ref. 15 in line 26 of page 3 should be ref. 14; ref. 16 in line 34 of page 3 should be ref. 15,16; “1981” in line 4 of Section 4 should be “1971”.

1. Original: The actinomycetes, particularly species from the genus *Streptomyces*, have proved to be a tremendous high-impact source of valuable chemicals. They have yielded many clinically essential antimicrobial compounds, including streptomycin, actinomycin, and streptothricin [8].

Revised: The actinomycetes, particularly species from the genus *Streptomyces*, have proved to be a tremendous high-impact source of valuable chemicals. They have yielded many clinically essential antimicrobial compounds, including streptomycin, actinomycin, and streptothricin [9].

2. Original: Incidentally, salinosporamide A, which holds promise for development of an anticancer drug, is produced by a strain of the genus *Salinispora,* a rare actinomycete isolated from a heat-treated marine sediment sample [9].

Revised: Incidentally, salinosporamide A, which holds promise for development of an anticancer drug, is produced by a strain of the genus *Salinispora*, a rare actinomycete isolated from a heat-treated marine sediment sample [10].

3. Original: It is believed that the actinomycetes are the source of some 61% of all microorganism-derived bioactive substances so far discovered [10].

Revised: It is believed that the actinomycetes are the source of some 61% of all microorganism-derived bioactive substances so far discovered [11].

4. Original: This suggests that rare actinomycetes are a valuable source of novel compounds, and that improved isolation strategies are required to increase the frequency in which they are isolated [11].

Revised: This suggests that rare actinomycetes are a valuable source of novel compounds, and that improved isolation strategies are required to increase the frequency in which they are isolated.

5. Original: Decades of success in our exploration of the actinomycetes is exemplified by the discovery in the Kitasato Institute by Satoshi Ōmura in the early-1970s of *Streptomyces avermectinius* (synonym *S. avermitilis*) MA-4680^T^, the microbe which produces the avermectins [12–14].

Revised: Decades of success in our exploration of the actinomycetes is exemplified by the discovery in the Kitasato Institute by Satoshi Ōmura in the early-1970s of *Streptomyces avermectinius* (synonym *S. avermitilis*) MA-4680^T^, the microbe which produces the avermectins [12,13].

6. Original: The avermectin derivative, ivermectin, is perhaps the world’s greatest, most effective, and safest drug for the treatment and prevention of a diverse range of human diseases and conditions [15].

Revised: The avermectin derivative, ivermectin, is perhaps the world’s greatest, most effective, and safest drug for the treatment and prevention of a diverse range of human diseases and conditions [14].

7. Original: The award citation stated “William C. Campbell and Satoshi Ōmura discovered a new drug, avermectin, the derivatives of which have radically lowered the incidence of River Blindness and Lymphatic Filariasis, as well as showing efficacy against an expanding number of other parasitic diseases” [16].

Revised: The award citation stated “William C. Campbell and Satoshi Ōmura discovered a new drug, avermectin, the derivatives of which have radically lowered the incidence of River Blindness and Lymphatic Filariasis, as well as showing efficacy against an expanding number of other parasitic diseases” [15,16].

8. Original: KML strain OS-3601 (Figure 1) was isolated from a soil sample collected at Aso, Kumamoto prefecture, Japan, in 1981,

Revised: KML strain OS-3601 (Figure 1) was isolated from a soil sample collected at Aso, Kumamoto prefecture, Japan, in 1971,

## 2. Change in Figure 2c

We would like to correct the structure of nanaomycin H as follow:

Original:
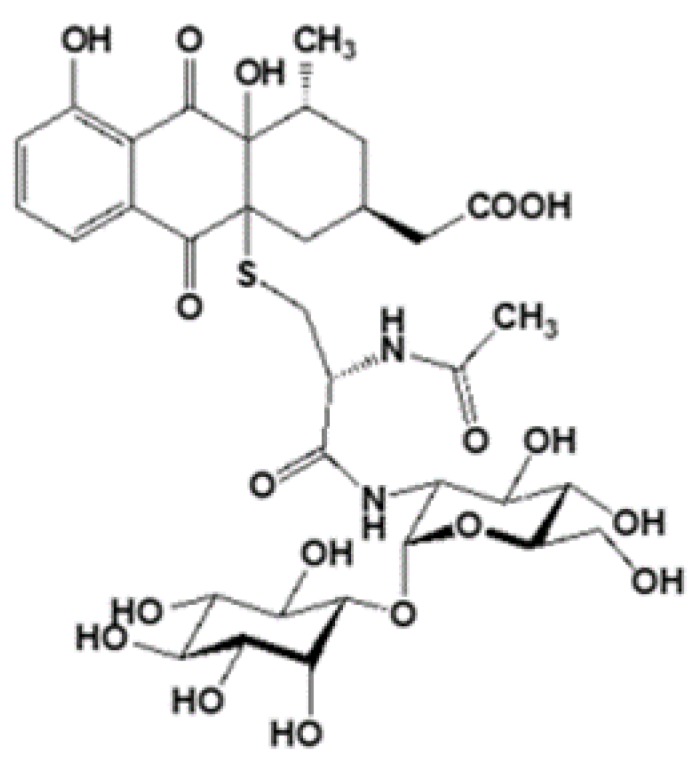
revised:
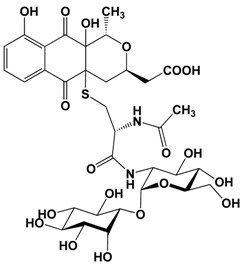


## 3. Changes in References

We would like to modify some references as follows:

Previous ref. 14 becomes ref. 12; previous ref. 12 is deleted; previous ref. 15 becomes ref. 14; ref. 15 is added as a new one.

The author would like to apologize for any inconvenience caused to the readers by these changes. The changes do not affect the major conclusions of the article. The manuscript will be updated, and the original will remain online on the article webpage.

## References

[1] Yōko T., Takuji N. Actinomycetes, an Inexhaustible Source of Naturally Occurring Antibiotics. Antibiotics.

